# Are Critical Fluctuations Responsible for Glass Formation?

**DOI:** 10.3390/ma17143385

**Published:** 2024-07-09

**Authors:** Szymon Starzonek, Joanna Łoś, Sylwester J. Rzoska, Aleksandra Drozd-Rzoska, Aleš Iglič

**Affiliations:** 1 Laboratory of Physics, Faculty of Electrical Engineering, University of Ljubljana, 1000 Ljubljana, Slovenia; ales.iglic@fe.uni-lj.si; 2 X-PressMatter Laboratory, Institute of High Pressure Physics of the Polish Academy of Sciences, 01-142 Warsaw, Poland; joalos@unipress.waw.pl (J.Ł.); sylwester.rzoska@unipress.waw.pl (S.J.R.); drozdrzoska@unipress.waw.pl (A.D.-R.)

**Keywords:** glass, critical phenomena, phase transitions, liquid crystals

## Abstract

The dynamic heterogeneities occurring just before the transition to the glassy phase have been named as the cause of amorphization in supercooled systems. Numerous studies conducted so far have confirmed this hypothesis, and based on it, a widely accepted solution to the puzzle of glass transition has been developed. This report focuses on verifying the existence of a strong pretransitional anomaly near the glass transition $T_{g}$. For this purpose, supercooled liquid-crystalline systems with a strong rod-like structure were selected. Based on the obtained experimental data, we demonstrate in this article that the previously postulated dynamic heterogeneities exhibit a critical characteristic, meaning a strong pretransitional anomaly can be observed with the described critical exponent α=0.5. Due to this property, it can be concluded that these heterogeneities are critical fluctuations, and consequently, the transition to the glassy state can be described based on the theory of critical phenomena. To measure the pretransitional anomaly near $T_{g}$ in supercooled liquid-crystalline systems, broadband dielectric spectroscopy (BDS) and nonlinear dielectric effect (NDE) methods were applied. The exponent α provides insight into the nature and intensity of critical fluctuations in the system. A value of α=0.5 suggests that the fluctuations become increasingly intense as the system approaches the critical point, contributing to the divergence in specific heat. Understanding the role of critical fluctuations in the glass transition is crucial for innovating and improving a wide range of materials for energy storage, materials design, biomedical applications, food preservation, and environmental sustainability. These advancements can lead to materials with superior properties, optimized manufacturing processes, and applications that meet the demands of modern technology and sustainability challenges.

## 1. Introduction

The theory of critical phenomena has found applications in various scientific fields, from physics to social sciences and economics. This is due to its universality and unique perspective on the analyzed problem. Previous research results have suggested that dynamic heterogeneities may be responsible for the transition to the glassy state, but there has been a lack of definitive evidence in materials and physical sciences. In this work, we aim to fill this gap and apply a critical-like description to one of the biggest mysteries of the 21st century: the glass transition.

For decades, understanding and explaining the nature of glass transition physics has been one of the great challenges of science. However, despite the elapsed time and the enormous number of experimental and theoretical studies, the long-awaited breakthrough in knowledge has not yet occurred [1,2,3,4,5,6]. When the glass temperature $T_{g}$ is exceeded, the transformation from a supercooled liquid to an amorphous solid glass state takes place. This transformation is ‘fuzzy’, i.e., it takes place over a range of temperatures. It is preceded by long-range changes in dynamic properties, such as the primary (alpha) relaxation time or viscosity, which show striking similarities across microscopically different systems [6].

It should be noted that solidification associated with melting/freezing and discontinuous phase transition occurs at specific temperatures, and no pretransition effects are expected in the liquid phase [7,8]. The exception is in systems with specific melting/freezing associated only with single symmetry elements, such as rod-like liquid-crystalline compounds [8,9,10,11,12,13] or orientationally disordered crystals [14,15,16,17], where critical long-range pretransitional effects occur. Remarkably, they are demonstrated not only in dynamic properties but also in static and thermodynamic properties.

This report shows evidence of critical-like and long-range pretransitional effects prior to the glass transition on cooling. They were obtained in rod-like nematogenic liquid-crystalline (LC) compounds that can vitrify at $T_{g}$ for any cooling rate. The evidence is related to the dielectric properties, including such basic static properties as the dielectric constant.

The characteristic feature of the glass transition is the predominant changes in the primary relaxation time $\tau (T\to T_{g})$ or viscosity $\eta (T\to T_{g})$, which start as early as 100–200 K above $T_{g}$. They are usually considered in the framework of the super-Arrhenius (SA) pattern [18,19]:

(1) $$\tau \left(t\right)=\tau_{\infty }\exp\left(\frac{E_{a}\left(T\right)}{RT}\right)$$

where $E_{a}\left(T\right)$ is the apparent, temperature-dependent activation energy, *R* stands for the gas constant, and $T=T_{g}$. For $E_{a}\left(T\right)=E_{a}const$, the classical Arrhenius (A) equation is obtained within a given temperature range.

Austin Angell [2,20,21,22,23,24,25] proposed the normalized plot that provides a uniform representation of previtreous changes for different glass formers—the $\log_{10}\tau \left(T\right)$ or $\log_{10}\eta \left(T\right)$ versus $T_{g}T$ plot—which became the hallmark of the mystery and challenge surrounding the glass transition. Important to its success was the empirical condition $\tau (T_{g})100$ s, which, for many low-molecular-weight glass-forming liquids, correlates with the glass temperature determined in the heat capacity scan for a cooling rate of 10 K/min. Another factor in the success of the Angell plot was the concept of fragility as a metric defining the degree of SA dynamics: $m{\left[d\log_{10}\tau \left(T\right)/d\left(T_{g}/T\right)\right]}_{TT_{g}}$. Fragility has become a central issue in glass transition physics, as it relates to its universalistic metric meaning [2,3,4,18,19].

The SA Equation cannot be used to represent experimental data because of the unknown form of the apparent activation energy. Therefore, substitute model scaling equations are used. The predominant position reached is the Vogel–Fulcher–Tammann (VFT) dependence [22,26,27,28]:

(2) $$\tau \left(T\right)=\tau_{\infty }\exp\left(\frac{A}{T-T_{0}}\right)=\tau_{\infty }\exp\left(\frac{D_{T}T_{0}}{T-T_{0}}\right)$$

where $T>T_{g}$ and $T_{0}<T_{g}$ represent the extrapolated VFT singular temperature and $D_{T}$ is the fragility strength coefficient associated with the fragility parameter.

Notwithstanding the distortion-sensitive analysis, we explicitly show that the adequacy of the VFT relation to represent τ(T) or η(T) experimental data is limited to only a limited number of glass formers, despite its enormous popularity [29]. It was one of the inspirations for the emergence of alternative scaling equations, which visual or residual tests indicated provided a similar quality of fit to the VFT dependence. The critical-like description of the glass transition addresses these limitations of the VFT model by providing a theoretical foundation based on the principles of critical phenomena, avoiding unphysical predictions, and explicitly accounting for dynamic heterogeneities. This approach offers deeper insights into the glass transition and enhances the predictive power for the behavior of supercooled liquids and glassy materials. However, a recent analysis of bias dependence has shown that only two equations passed validation tests across a wider group of different glass formers [29]. These are the MYEGA equation of the SA type [30] and the recently proposed active-critical equation, i.e., the equation containing the SA type and critical terms [31]. The latter has the form [29,31]:

(3) $$\tau \left(T\right)=C_{\Gamma }{\left[t^{-1}\exp\left(t\right)\right]}^{\Gamma }$$

where $t=(T-T^{*})T$ and $T^{*}=T_{g}$ represent the extrapolated singular temperature. An exception is glass formers composed of molecules with predominant uniaxial symmetry, where the critical representation provides a similar quality of fit [29,32,33]:

(4) $$\tau \left(T\right)=\tau_{0}{\left(T-T^{*}\right)}^{-\phi }$$

This result was supported by enthalpy-space, distortion-sensitive analysis, which explicitly showed prevalence over the VFT portrayal [32,33,34]. Notably, Equation (4) with the exponent ϕ=9 was derived within the semi-empirical dynamical scaling model (DSM) by Colby and Erwin [35,36]. The authors suggested its general validity for low-molecular-weight and polymeric glass formers, which was supported by a table showing validation across 43 different glass formers. However, external check tests did not confirm the validity of the DSM portrayal. Later, the authors of this report demonstrated that the critical-like portrayal (Equation (4)) with the exponent ϕ≈9 for $T\to T_{g}$ applies to glass formers with a uniaxial molecular structure.

The glass transition is considered an exceptional state transformation with a dynamic nature, which is supported by the above-mentioned extraordinary previtreous changes. It is also related to some $T_{g}$ shifts for different cooling rates. The unique dynamic nature of the glass transition also stresses the lack of previtreous anomalies for non-dynamic physical properties. The leading example may be the heat capacity, which shows some characteristic changes close to $T_{g}$, but they are diverse, and their universalistic parameterization seems to be impossible [4,29,37].

Notwithstanding this, the following relation has recently been proven for large-scale changes in configurational entropy, describing the excess entropy in a supercooled fluid in non-equilibrium [38]:

(5) $$S_{c}\left(T\right)=S_{0}{\left(1-\frac{T^{*}}{T}\right)}^{n}$$

where $T=T_{g}$ and the extrapolated singular temperature $T^{*}=T_{g}$ coincides with Kauzmann’s ‘critical’ temperature of the ideal glass, i.e., $S_{c}\left(T\to T_{K}\right)\to 0$ and $T^{*}\approx T_{K}$. Values of 0.16<n<1 were obtained for systems with 3D positional symmetry, e.g., orientationally disordered crystals (ODICs), and 1.6>n>1 for systems with dominant uniaxial molecular symmetry, as in glass, which is almost a rod-like LC. Based on these results, the relation for previtreous changes of the configurational contribution to the heat capacity at constant pressure was derived [38]:

(6) $$\Delta C_{conf}\left(T\right)=C_{0}{\left(1-\frac{T^{*}}{T}\right)}^{n-1}$$

Good agreement with experimental data, i.e., reproduction of observed heat capacity patterns of previtreous changes, has been shown [38]. It is noteworthy that Equation (6) and the mentioned values of the exponent n were obtained using ‘dynamic’ values of τ(T) experimental data transformed into the apparent activation energy index $I_{DO}\left(T\right)=\left[\left(d\ln E_{a}\left(T\right)\right)d\ln T\right]$ using Equation (1) and the Adam–Gibbs (AG) glass transition model that links τ(T) and $S_{c}\left(T\right)$ [39]. With respect to the Adam–Gibbs model, the concept of cooperatively rearranged regions (CRRs) changing the configurational entropy was introduced, leading to the following model relationship for the primary relaxation time previtreous changes [40,41]:

(7) $$\tau =\tau_{\infty }\exp\left(\frac{A_{AG}}{TS_{c}\left(T\right)}\right)$$

where $T>T_{g}$ and $A_{AG}$ is the AG model-related constant.

In the isotropic liquid phase, glass behaves as a highly viscous liquid. Its viscosity decreases with increasing temperature. This behavior is typical of supercooled liquids, where viscosity can change over many orders of magnitude with a relatively small change in temperature. The flow behavior follows Newtonian flow at higher temperatures, where the stress is directly proportional to the strain rate. However, as the temperature decreases, the glass may exhibit non-Newtonian behavior, where the relationship between the stress and the strain rate becomes nonlinear.

The viscosity of glass in this phase follows the Arrhenius equation at high temperatures but deviates at lower temperatures, often modeled using the Vogel–Fulcher–Tammann (VFT) equation. This describes how the viscosity exponentially increases as the temperature approaches the glass transition temperature $T_{g}$. As the temperature approaches $T_{g}$, the glass transitions from a viscous liquid to a glassy state. In the isotropic liquid phase, the system has not yet reached this transition, but it is close, and molecular mobility significantly decreases, resulting in a dramatic increase in viscosity. Rod-like structures may introduce anisotropy in the isotropic liquid. This means that the viscosity may vary depending on the direction of flow relative to the alignment of the rods. If the rods tend to align in a particular direction due to flow, the system can exhibit lower viscosity in the direction of alignment and higher viscosity perpendicular to it.

This report presents studies of the dielectric constant and curves associated with the loss maximum in three glass-forming rod-like (uniaxial) liquid-crystalline (LC) compounds. These are isopentylcynobiphenyl (5*CB), isooctyloxycyanobiphenyl (8*OCB), and a eutectic mixture of four LC compounds: E7. The latter vitrifies on the passage of $T_{g}$ from the nematic phase (N), 8*OCB from the isotropic liquid phase (I), and 5*OCB from the chiral nematic (N*). Nevertheless, in each case, there is evidence for the previtreous behavior of the dielectric properties mentioned. This anomaly parallels the pretransitional effect observed when cooling the isotropic liquid phase toward I-N transitions, which is explicitly associated with fluctuations of the subsequent mesophase.

## 2. Materials and Methods

### 2.1. Materials

(S)-4-(2-methylbutyl)-4′-cyanobiphenyl (5*CB) [42,43,44,45,46], (S)-4-(1-methylheptyloxy)-4′-cyanobiphenyl (8*OCB) [46,47], and the eutectic mixture E7 [24,25,48,49] were investigated. The latter has the following composition: 5CB (51%), 7CB (25%), 8OCB (16%), and 5CT (8%). They exhibit an uncommon property among rod-like LC compounds, namely supercooling to $T_{g}$ at any cooling rate in practice. Consequently, they are characterized by the following phase sequences:

5*CB: isotropic liquid → chiral nematic (N*) → glass

8*OCB: isotropic liquid → glass

E7: isotropic liquid → nematic (N) → glass

It is noteworthy that 5*CB and 8*OCB are isomers of the ’classical’ rod-like nematogenic compounds with well-known mesomorphism. The first criterion for selecting 5*CB, 8*OCB, and E7 is their ability to be supercooled. Additionally, E7 allows for obtaining a supercooled nematic phase, 5*CB a supercooled chiral phase, and 8*OCB supercools in the isotropic phase. Such a selection of materials enables the assessment of the occurrence of critical fluctuations with different symmetries within the studied systems. The second equally important criterion for choosing these materials is the very rich base of available references.

E7 was acquired from Synthon GmbH and thoroughly purified. 5*CB and 8*OCB were provided courtesy of Krzysztof Czupryński (Military Technical University, Warsaw, Poland).

### 2.2. Methodology

The measurements of the complex dielectric permittivity $\varepsilon^{*}\left(\omega \right)=\varepsilon^{\prime }\left(\omega \right)+i\varepsilon^{\prime \prime }\left(\omega \right)$, where (ω=2πf), were performed using BDS Novocontrol (Montabaur, Germany) impedance analyzer, supported by a Quattro temperature control unit with temperature stability better than ΔT=0.2 K. The samples were placed in a flat-parallel capacitor with a diameter of 2r=20 mm and a gap of d=0.3 mm to maintain bulk behavior during the experiments. The applied voltage was U=1 V, which is typical for a linear dielectric response.

**Figure 1:**
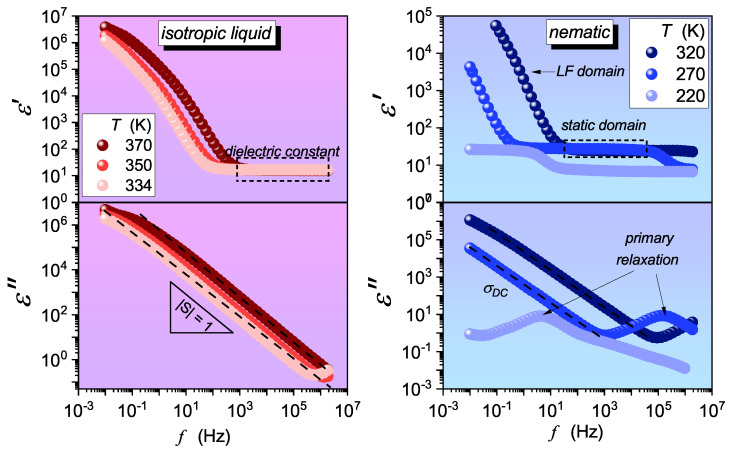
Examples of experimentally determined complex dielectric permittivity spectra in the isotropic liquid and nematic LC phases of E7. Characteristic features, such as the static range, coupled with the dielectric constant, DC conductivity DC, or the primary relaxation time loss curves, are shown. Note the shift in the static domain during cooling toward the glass temperature.

For 5*CB and 8*OCB, we investigated a new problem—pretransitional behavior approaching the glass transition temperature $T_{g}$—which is hidden in the complex dielectric spectra that formed the basis of previous studies by the authors of [31,43,44,47]. New measurements were performed for the E7 mixture. The master plot showing the obtained complex dielectric permittivity spectra is shown in Figure 1.

In general, in rod-like LC compounds, the canonical primary (alpha) relaxation process via the imaginary part of complex electric permittivity, $\varepsilon^{\prime \prime }\left(f\right)$, is observed only in the isotropic liquid phase. In the nematic phase, the relaxation often splits into the dominant δ-process associated with the long molecular axis and a much lower γ-process located at higher frequencies, associated with the short molecular axis orientation. In this report, the α-process is considered in the nematic phase and the δ-process as its successor in the nematic mesophase. The peak of the primary loss curve is characterized by the maximum ($\varepsilon_{max}^{\prime \prime }$) and the peak frequency ($f_{peak}$), which define the primary relaxation time.

### 2.3. Data Analysis

The obtained data of the complex electric permittivity $\varepsilon^{*}\left(\omega \right)$ were analyzed as follows. To determine the phase transitions, we used the dielectric constant $\varepsilon_{s}$, which is the value of the real part of the complex electric permittivity occurring typically between $10^{7}$ and $10^{3}$ Hz (Figure 1) for the isotropic liquid phase and $10^{5}$ and $10^{1}$ for the mesophases [9,10,11,12,13,14,15,16,17,18]. These frequency ranges exhibit a plateau value of $\varepsilon^{\prime }\left(\omega \right)$, which is related to the total dipole polarization of the sample—the static domain [9,10,11,12,13,14,15,16,17,18]. By portraying the temperature dependence of the dielectric constant, one can detect phase transitions as discontinuities or continuities in $\varepsilon_{s}\left(T\right)$. It is worth noting that the critical description of $\varepsilon_{s}\left(T\right)$ refers to a critical exponent α, which is related to the heat capacity behavior ($C_{p}$) near the phase transition temperature [9,10,11,12,13,14].

The primary relaxation time occurring on the imaginary part of the complex electric permittivity spectra can be defined as $\tau =\tau_{\alpha }=1/\left(2\pi f_{peak}\right)$. However, from a practical point of view, it is more popular to analyze dispersion spectra using the Havrilliak–Negami function [18,19].

To analyze all obtained data, the following software was used: WinDETA (Novocontrol, Montabaur, Germany), WinFIT (Novocontrol, Montabaur, Germany), and OriginPro 2018 (OriginLab).

## 3. Results and Discussion

### 3.1. Previtreous Behavior of the Primary Relaxation Time

**Figure 2:**
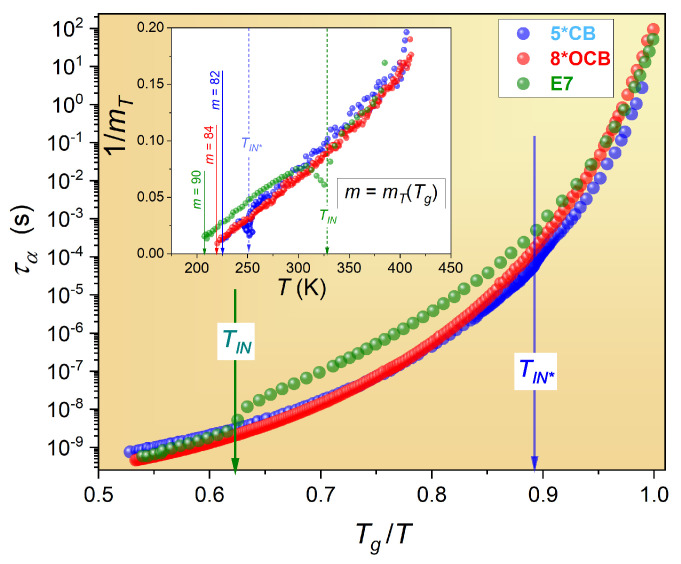
Angell plot showing the previtreous changes in the primary relaxation time for 5*OCB, 8*OCB, and E7 liquid-crystalline glass-forming systems. It is related to $T_{g}=223.15$ K (5*CB), $T_{g}=220.3$ K (8*OCB), and $T_{g}=208.3$ K (E7). The inset shows the reciprocal of the evolution of the apparent fragility, $m_{T}$, i.e., the first derivative of the experimental data from the main part of the graph (Equation (8)). The vertical dashed arrows in the inset show the phase transitions in 5*CB (I-N*) and E7 (I-N). The value of the fragility parameter $m_{T}$ at $T=T_{g}$ is indicated by an arrow.

Figure 2 shows the evolution of the primary relaxation time on the Arrhenius scale using the normalized Angell plot, showing the previous changes in the primary relaxation time for 5*CB, 8*OCB, and E7. The inset shows the changes in apparent fragility for $T\geq T_{g}$, derived from the experimental data in the main part of Figure 2:

(8) $$m_{T}=\left[\frac{d\log_{10}\tau \left(T\right)}{d(T_{g}/T)}\right]$$

From the above relationship, the fragility parameter $m=m_{T}\left(T_{g}\right)$ is obtained. These values are given in Figure 2. The inset in Figure 2 shows the linear behavior of the apparent fragility coefficients extending even up to 100 K above $T_{g}$. This behavior confirms the following pattern of changes [31]:

(9) $$m_{T}\left(T\right)\propto \frac{1}{T-T^{*}}$$

where $T^{*}$ is the extrapolated singular temperature obtained from the linear regression analysis (see inset), or the condition $m_{T}\left(T^{*}\right)=0$.

The analysis of the apparent fragility shown in Figure 2 reveals that $T^{*}$(5*CB) = 213.8 K, $T^{*}$(8*OCB) = 206.5 K, and $T^{*}$(E7) = 187.5 K. Knowing the values of the glass temperature, one can consider the metric of the glass transition discontinuity, $\Delta T_{g}^{*}=T_{g}-T^{*}$, which is 9.3 K for 5*CB, 13.8 K for 8*OCB, and 20.8 K for E7. In Ref. [31], Equation (9) was validated as an empirical universal scaling relation based on the analysis of a series of microscopically different glass formers. It was subsequently used to derive the active-critical Equation (3) for τ(T) previtreous changes.

The Angell plot and apparent fragility analysis have limitations in representing previtreous changes in the primary relaxation time for different glass formers due to several factors. The normalization and scaling used in Angell plots, typically $T_{g}/T$, can obscure absolute temperature effects and might not accurately represent the dynamics of glass formers with widely varying $T_{g}$ values. Additionally, these plots focus predominantly on the behavior near the glass transition temperature, potentially overlooking significant dynamics at higher temperatures where previtreous changes occur. The non-universal behavior highlighted by the strong-fragile distinction may not fully capture the continuum of relaxation behaviors seen in various materials. Moreover, the empirical nature of these plots makes it challenging to derive universal principles or predictive models for different glass formers, limiting their utility in fully characterizing the complex relaxation processes that occur in the previtreous region.

**Figure 3:**
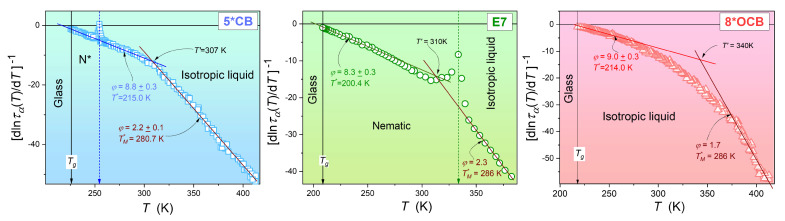
The derivative-based investigation related to Equation (6), focused on the validation of the critical-like Equation (5) in isopentylcyanobiphenyl (5*CB). The values of the relevant parameters of Equations (5) and (6) are given.

Figure 3 shows the results of the derivative-based and bias-sensitive test, which focuses on validating the description of the experimental data of τ(T) using the critical-like Equation (4). It is based on the proposed analytical transformation of the experimental data as follows:

(10) $$\tau_{\alpha }\left(T\right)=\tau_{0}{\left(T-T^{*}\right)}^{-\phi }\to \ln\tau_{\alpha }=\ln\tau_{0}-\phi \ln\left(T-T^{*}\right)\to {\left[\frac{d\ln\tau_{\alpha }\left(T\right)}{dT}\right]}^{-1}=-\phi \left(T-T^{*}\right)$$

where $\tau_{0}$ is a theoretical value of the relaxation time at high temperatures, $T^{*}$ describes the temperature where a hypothetical continuous phase transition takes place, and ϕ is a critical-like exponent.

The results shown in Figure 3 reveal two critical domains in each glass former. The first is near the glass temperature up to about $T_{g}=50$ K. It is associated with the exponent ϕ≈9 and the values of the extrapolated singular temperatures $T^{*}$, which are similar to those obtained from the apparent fragility analysis in Figure 2. It is noteworthy that the I-N or I-N* phase transition causes only local distortions in the dominant pattern of τ(T) changes.

The critical domains near the glass transition temperature $T_{g}$ and in the high-temperature region correlate well with the predictions of mode-coupling theory (MCT). Near $T_{g}$, MCT predicts a significant slowing down of dynamics as the temperature approaches a critical value $T_{c}$, leading to a divergence of relaxation times and the onset of dynamic heterogeneity, which aligns with the observed critical domain where relaxation times increase sharply, indicating enhanced molecular cooperativity. In the high-temperature region, MCT describes an initial regime where the system’s dynamics are dominated by caging effects, leading to a plateau in the relaxation time before the onset of slower dynamics as the system cools down, correlating with the critical domain observed at higher temperatures, where relaxation times begin to deviate from simple Arrhenius behavior and show more complex dependencies, reflective of the evolving intermolecular interactions and structural rearrangements.

**Figure 4:**
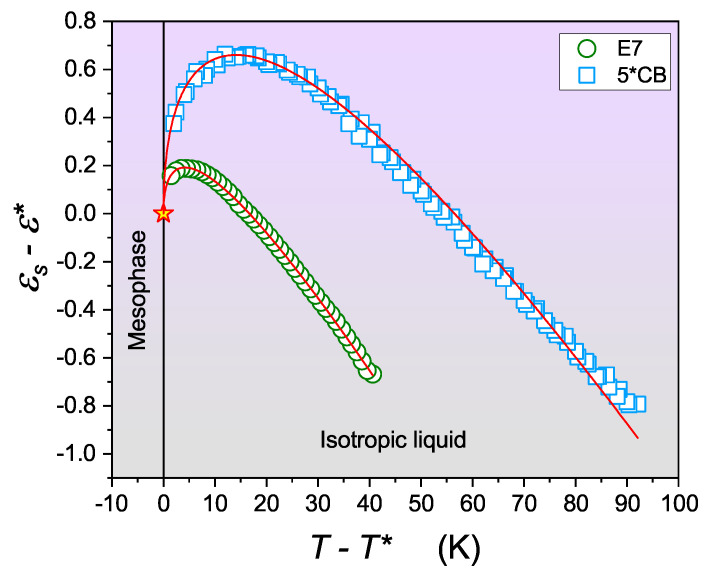
Pretransitional anomaly of dielectric constant for the iso–mesophase transition, presented in reduced scale for E7 and 5*CB. The red star denotes the point where the ideal continuous phase transition takes place.

**Figure 5:**
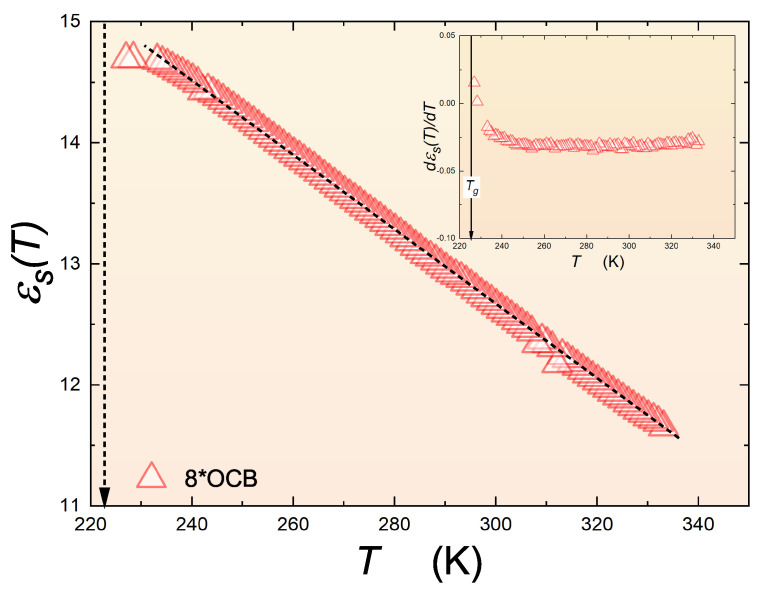
Changes in the dielectric constant in the isotropic liquid phase of 8*OCB during cooling to the glass temperature, indicated by the solid arrow. The inset shows the derivative of the data from the main part of the graph.

The analysis presented in Figure 3, Figure 4 and Figure 5 shows a second critical-like region in the high-temperature region, characterized by the critical-like relation with the extrapolated singular temperature $T_{M}^{*}>T_{g}$ and power exponent ϕ≈2. Such behavior is consistent with general expectations for any glass-forming system in a high-temperature regime and is supported by the predictions of mode-coupling theory (MCT) [37,44]. The difference in the temperature evolution of the primary relaxation time or coupled dynamical quantities near $T_{g}$ and far from $T_{g}$ is consistent with the partitioning of the supercooled, previtreous region into a non-ergodic and an ergodic dynamical region. Remarkably, Figure 3, Figure 4 and Figure 5 show that the high-temperature ergodic domain can seamlessly transition from the supercooled non-equilibrium region to the ‘distant’ equilibrium domain, i.e., the ‘normal’ liquid state.

### 3.2. Pretranstional Anomalies in the Isotropic Phase

Understanding the pretransitional anomalies in the isotropic phase of liquid-crystalline materials has significantly broader implications for both the scientific understanding and practical applications of these materials. Scientifically, it provides deeper insights into the fundamental nature of phase transitions and critical phenomena, enhancing our knowledge of how molecular interactions and fluctuations evolve near transition points. This understanding can help refine theoretical models, such as mode-coupling theory and mean-field approximations, making them more accurate for predicting material behavior under various conditions.

Practically, the ability to predict and control pretransitional behavior is crucial for the development of advanced liquid-crystal technologies. For instance, it can lead to improved performance and novel functionalities in liquid-crystal displays (LCDs), where precise control over the alignment and optical properties of the liquid crystal is essential. Additionally, understanding these anomalies can aid in the design of responsive materials for sensors and actuators, where the material’s properties need to change predictably in response to external stimuli. Furthermore, it can inform the development of new liquid-crystalline materials with tailored properties for specific applications, such as tunable photonic devices, adaptive lenses, and smart windows.

As the isotropic–nematic (I-N) phase transition temperature is approached, strong pretransitional effects are observed due to several specific physical mechanisms. These include enhanced molecular orientational correlations and fluctuations as molecules begin to align locally in response to thermal fluctuations, even before the bulk transition occurs. This pretransitional alignment leads to a gradual increase in the local order parameter and the formation of transient nematic domains within the isotropic phase. Additionally, the decrease in rotational entropy as molecules become more directionally constrained contributes to a rapid increase in viscosity and a divergence of relaxation times. The coupling between orientational and translational degrees of freedom also plays a role, leading to changes in the system’s dynamic properties, such as the diffusion coefficients. These pretransitional effects are often manifested in measurable physical properties like birefringence, susceptibility, and specific heat, reflecting the system’s approach toward the long-range orientational order characteristic of the nematic phase [7,8,9,10,11,12,13,14,15,16,17].

One of the fundamental physical properties that shows a change in the dielectric constant before the transition when cooling toward $T_{IN}$ has been demonstrated [24,25,47,48,49,50,51]:

(11) $$\varepsilon_{s}\left(T\right)=\varepsilon^{*}+a\left(T-T^{*}\right)+A{\left(T-T^{*}\right)}^{1-\alpha }$$

where $T>T^{C}$ and $T^{C}$ denotes the clearing temperature related to the isotropic liquid–LC transition; in the given case, $T^{C}=T_{IN}$. $T^{*}<T^{C}$ is the extrapolated temperature of a hypothetical continuous phase transition; $\varepsilon^{*},a,A$ are constant parameters, and the exponent α≈0.5 can be related to the heat capacity critical exponent within the mean-field or tricritical approximation [8]. The identification of the critical index α=0.5 in the glass transition provides significant insights into the nature of this transition. It suggests that the glass transition can be described using the framework of critical phenomena, with dynamic heterogeneities playing a crucial role as critical fluctuations. This aligns the glass transition with a broader class of critical behaviors observed in other phase transitions while also highlighting its unique characteristics due to the disordered and non-equilibrium nature of glassy systems. This understanding could pave the way for developing new theoretical models and improving material design by leveraging the critical-like properties of glass-forming systems.

Pretransitional effect studies allow the measurement of the phase transition discontinuity metric: $\Delta T^{*}=T^{C}-T^{*}$ [8]. In general, this significant feature is determined by different physical methods for the I-N transition: The obtained values $\Delta T^{*}=1-2$ K show its weakly discontinuous character [8,12,13,14,15,16,17]. However, the position of the dielectric constant is unique among physical quantities showing pretransitional effects in the isotropic liquid phase. Indeed, for the dielectric constant, Equation (11) also describes pretransitional effects in approaching the phase transition to chiral nematic (N*) [43,44], smectic A (SmA) [52], and smectic E (SmE) [53] LC mesophases. This results from the fact that the dielectric constant captures the arrangement of permanent dipole moments, which is the same across all mentioned LC mesophases. Consequently, the dielectric constant can serve as a universal tool for estimating $\Delta T^{*}$ for the phase transition to any LC mesophase. For I-SmA, where a one-dimensional translational arrangement exists in addition to the uniaxial nematic-type arrangement, a value of $\Delta T^{*}\approx 10$ K was determined. For the transition to SmE, where positional arrangement in addition to the uniaxial arrangement of the nematic type takes place, values of $\Delta T^{*}\approx 30$ K were found [53].

Figure 4 shows the pretransitional anomaly for the as-yet untested mixture E7 LC, which is well represented by Equation (11). The pretransitional behavior of the dielectric constant anomaly is related to the contribution of the pre-mesomorphic (prenematic) fluctuation in the isotropic liquid environment. The fundamental feature of the uniaxial order of the nematic type is the equivalence of the $\vec{n}$ and $-\vec{n}$ directors, which indicate the average direction of the long axis or rod-like molecules. This leads to the cancellation of the dipolar contribution to the dielectric constant within the fluctuations before the transition. Consequently, the dielectric constant associated with the fluctuations is much lower than in the isotropic liquid environment. However, the correlation length (ξ) increases during cooling, which increases its volume ($V_{fluct}$) even more, as follows [8]:

(12) $$\xi \left(T\right)=\xi_{0}{\left(T-T^{*}\right)}^{-\nu }=\xi_{0}{\left(T-T^{*}\right)}^{-0.5}\to V_{fluct}∼{\left(T-T^{*}\right)}^{-1.5}$$

The interplay between the contributions of prenematic fluctuations and the isotropic liquid environment manifests in the following crossover for the pretransitional effect: $d\varepsilon_{s}\left(T\right)/dT<0\to d\varepsilon_{s}\left(T\right)/dT>0$, i.e., from the dominance of the ‘parallel’ arrangement of permanent dipole moments to the ‘antiparallel’ arrangement. Moreover, prenematic fluctuations refer to the local, transient alignments of molecules that occur even before the bulk nematic phase forms. These fluctuations increase as the temperature approaches the transition point, leading to a gradual rise in the local order parameter within the isotropic phase. The isotropic liquid environment, characterized by random molecular orientations and higher entropy, moderates these prenematic fluctuations, preventing immediate long-range order but allowing short-range correlations to grow. The balance between these two factors results in a variety of observable pretransitional effects, such as an increase in the dielectric constant, susceptibility, and viscosity. The isotropic liquid environment ensures that these fluctuations remain dynamic and dispersed, rather than forming a stable nematic phase, which only solidifies once the critical transition temperature is crossed. This dynamic interplay contributes to the continuous and smooth nature of the phase transition, with prenematic fluctuations enhancing local ordering and the isotropic environment providing a medium for these fluctuations to manifest without leading to an abrupt phase change [51,52,53].

Refs. [54,55,56] show that the maximum of the $\varepsilon^{\prime \prime }\left(f\right)$ loss curve in isotropic or rod-like LC materials shows temperature changes parallel to Equation (11), as follows:

(13) $$\varepsilon_{peak}^{\prime \prime }\left(T\right)=\varepsilon_{peak}^{*}+a_{peak}\left(T-T^{*}\right)+A_{peak}{\left(T-T^{*}\right)}^{1-\alpha }$$

It is noteworthy that the tests of the dielectric constant in the isotropic liquid, related to Equation (11), are associated with constant frequency scans of $\varepsilon^{\prime }$ in the static region, typically around f≈10–100 kHz in the given case. The evolution of the primary loss curve in the isotropic liquid phase is associated with variable frequencies. For example, in classical nematogenic LC materials such as 5CB, these frequencies change from f≈ 600 MHz at $T_{IN}50$ K to f≈2 MHz just above $T_{IN}$. Experimental investigations have shown that analyzing the experimental data via Equation (11) for τ(T) and via Equation (13) for $\varepsilon_{peak}^{\prime \prime }$ yield the same values for the exponent α≈0.5 and the discontinuity $\Delta T^{*}$ [54,55,56].

In discussing the origins of Equation (13), it is worth noting the considerations recently presented in Ref. [56], which state that the maximum of the loss curve can be regarded as a measure of the energy required for reorientation associated with the primary (alpha) relaxation process. In the isotropic liquid phase, uniaxially ordered prenematic fluctuations occur in the isotropic liquid environment and form a specific ‘critical colloid’. The energy required for realignment in the two parts of this system must be different. The isotropic fluid component changes slowly and increases linearly as it cools. As described above, the fluctuations show critical type changes, leading to Equation (13).

In Refs. [52,53,54,55,56], the derivatives of the experimental data were additionally considered. With reference to Equations (11) and (13), they should show the following anomalies before the transition:

(14) $$\frac{d\varepsilon_{s}\left(T\right)}{dT}=a+A\left(1-\alpha \right){\left(T-T^{*}\right)}^{-\alpha }$$

(15) $$\frac{d\varepsilon_{peak}^{\prime \prime }\left(T\right)}{dT}=a_{peak}+b_{peak}{\left(T-T^{*}\right)}^{-\alpha }$$

The derivation of experimental data reduces the number of fitted parameters. Simultaneous analysis of $\varepsilon_{s}\left(T\right)$ and $d\varepsilon_{s}\left(T\right)/dT$ using Equations (11) and 14 or $\varepsilon_{peak}^{\prime \prime }\left(T\right)$ and $d\varepsilon_{peak}^{\prime \prime }\left(T\right)/dT$ supports the fitting. The above analysis was successfully performed on a number of LC compounds, including 5*CB [44] and the basic components of the E7 mixture: 5CB [55] and 8OCB [56]. The changes in the dielectric constant represented by Equations (11) and (14) extend even to 100 K above $T_{IN}$. The maximum of the loss curve described by Equations (13) and (15) ends at about 30–40 K above $T_{IN}$. The strength of the $\varepsilon_{peak}^{\prime \prime }\left(T\right)$ anomaly before the transition is larger than for $\varepsilon_{s}\left(T\right)$ [44,55,56]. It is noteworthy that 8*OCB supercools to the glass transition temperature in the isotropic liquid phase without pretransitional effects, as previously demonstrated [46,47,48].

### 3.3. Critical-like Behavior near the Glass Transition

In the tests performed so far on glass-forming liquids cooled to $T_{g}$, the dielectric constant $\varepsilon^{\prime }\left(f\right)$ for a selected frequency, usually *f* 1 MHz, is usually investigated [57,58,59]. These tests, with a constant cooling or heating rate similar to thermal DSC tests, effectively determine the glass transition temperature during cooling or the crystallization temperature that may occur when heating starting from the amorphous glass phase. These results have not yet shown the occurrence of a previtreous effect for $T\to T_{g}$. However, the measurements for $\varepsilon^{\prime }\left(f=1\;\mathrm{MHz}\right)$ cannot be correlated with the dielectric constant because the position of the static domain changes, as shown in the Methods section. There are also reports that focus on the dielectric strength $\Delta \varepsilon_{strength}=\varepsilon -\varepsilon_{\infty }$, where $\varepsilon_{\infty }$ is determined by the atomic (non-dipolar) polarizability [60,61]. The values of the strength are determined from model analysis of the primary loss curve $\varepsilon^{\prime \prime }\left(f\right)$ for subsequent temperatures using the Havriliak–Negami (HN) relation [60,61]. In this functionally complex relation, Δε is one of the five fitted parameters, which significantly limits the resolution of the obtained parameters.

Figure 5 shows the results of the measurement of the dielectric constant in the isotropic liquid phase, almost up to the glass temperature, in glass-forming 8*OCB. The dielectric constant was explicitly determined as $\varepsilon^{\prime }\left(f\right)$ within the middle of the static range to avoid distortions associated with the shift in the dielectric spectra toward $T_{g}$ during cooling. It is noteworthy that the experimental data end before reaching the glass transition temperature. This is related to the huge time-frequency shift as $T_{g}$ is approached, as can be seen in the discussion of the primary relaxation time above. Reaching the static domain scan in the immediate vicinity of $T_{g}$ requires $\varepsilon^{\prime }\left(f\right)$ to reach frequencies as low as $10^{-5}$ Hz, which is always (very) difficult experimentally. For the peak of the loss curve $\varepsilon_{peak}^{\prime \prime }$, the determination of the scan above $10^{-2}$ Hz is satisfactory. Notwithstanding, the decrease in $\varepsilon_{s}$ for $T\to T_{g}$ is visible below the linear pattern occurring far from $T_{g}$. The bias-sensitive derivative analysis of the experimental data from the main part of the plot reveals the pretransitional/previtreous effect starting as much as 40 K above $T_{g}$. The solid curve is correctly represented by Equation (14) with the exponent α=0.5. The parameters obtained enable the description of the experimental data of $\varepsilon_{s}$ over a long period of time, as shown by the solid curve. Note the inset below, which focuses on the behavior near $T_{g}$.

**Figure 6:**
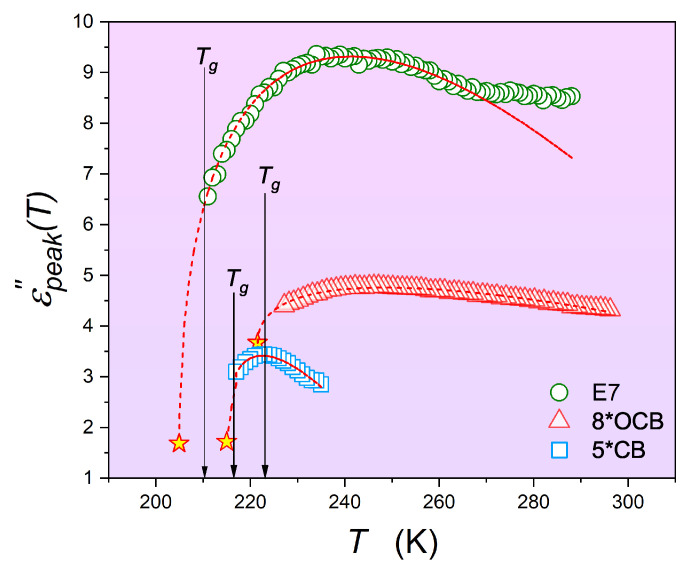
Changes in the maxima of the loss curves on cooling toward the glass transition in 8*OCB (isotropic liquid phase), 5*CB (chiral nematic phase), and the E7 mixture (nematic phase).

**Figure 7:**
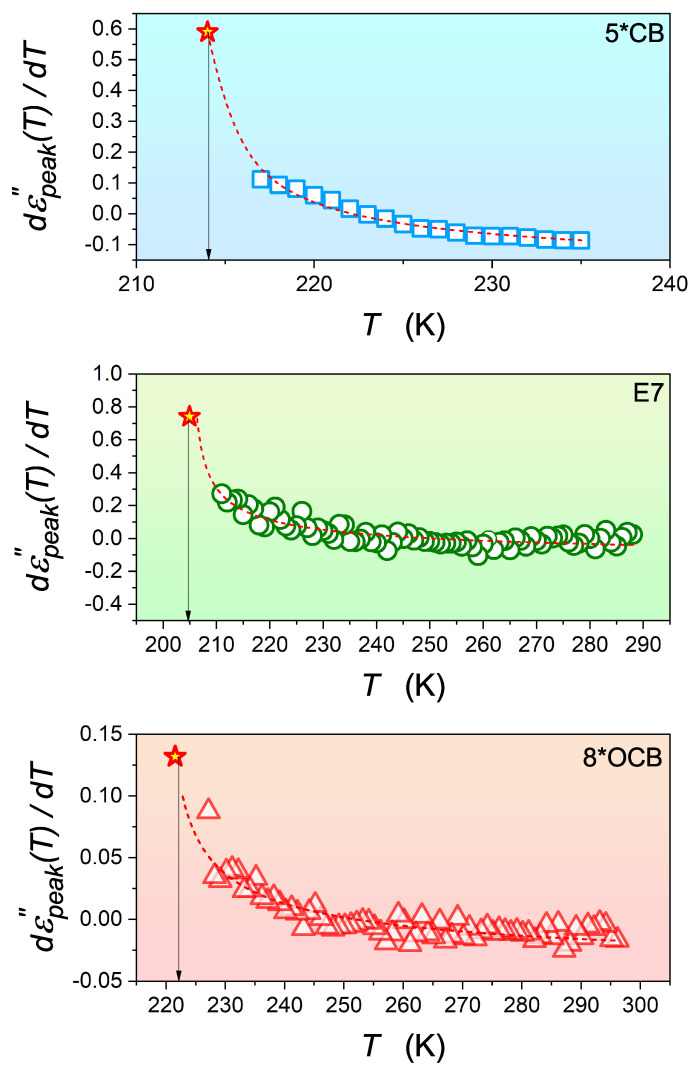
The representation of the derivatives of the maxima of the primary loss curves, showing a critical-like behavior described by Equation (15) with the exponent α=0.5. Arrows show the glass transition temperature $T_{g}$.

The nematic phase is most commonly tested for perpendicular and parallel alignment of rod-like molecules in two modes: (i) the long molecular axis and the intensity of the electric field of measurement are parallel, and (ii) the long molecular axis and the intensity of the electric field of measurement are perpendicular. The difference $\varepsilon_{\|}-\varepsilon_{⊥}$ is used as one of the order parameters for nematogenic LC [8]. For non-oriented samples, as in the present report and all measurements performed so far on glass-forming LC compounds, one can obtain arbitrary patterns of changes between $\varepsilon_{\|}\left(T\right)$ and $\varepsilon_{⊥}\left(T\right)$. Consequently, a reliable discussion of the dielectric constant in the nematic phase, for example, in 5*CB or 8*OCB, is not possible when cooling to $T_{g}$. However, one can focus on $\varepsilon_{peak}^{\prime \prime }\left(T\to T_{g}\right)$, where the above limitation is not important, especially when examining previtreous/pretransitional effects in the LC systems discussed in this report. These results are shown in Figure 6 and Figure 7.

Figure 6 and Figure 7 also show strong and extensive pretransitional (previtreous) effects for 5*CB, E7, and 8*OCB. The solid curves show that Equations (13) and (15) with the same exponent α=0.5 can represent these well. Note the small values of the ‘discontinuities’ $\Delta T_{g}^{*}=T_{g}-T^{*}$, similar to those obtained for the I-N transition.

For comments on the results presented above, the discussion of Equation (11) may be relevant, as it is the basic reference for the I-N transition. Thoen and Menu [50] and Drozd-Rzoska et al. [51] introduced this dependence by using the parallel to $\varepsilon_{s}\left(T\right)$ precritical changes in critical binary mixtures. This somewhat heuristic approach was complemented [51,54,55,56] by linking it to the model consideration of Mistura [62], who showed the direct relationship between the heat capacity and dielectric constant $C_{p}\propto d\varepsilon_{s}\left(T\right)/dT\propto {\left(T-T_{C}\right)}^{-\alpha }$ in the supercritical region. As mentioned earlier, this prediction was confirmed by authors A.D.R. and S.J.R. for critical mixtures with limited miscibility [63,64,65,66,67,68,69,70] and the I-N transition in LC materials [17,18,19,55,56]. Sengers et al. [71,72] derived Equation (11) for $\varepsilon_{s}\left(T\right)$ anomalies when approaching the critical temperature $T_{C}$ in binary critical mixtures and for the gas–liquid critical point in single-component systems. It was determined using a thermodynamic model that takes into account entropy and internal energy changes influenced and detected by a weak external electric field.

To describe pretransitional anomalies in the isotropic phase of nematogenic LC and the liquid phase of orientationally disordered crystals based on the dielectric constant and its extension associated with a strong electric field, the nonlinear dielectric effect (NDE) can be applied. This reasoning is reminiscent of the model relationship that has been successfully used to explain the precritical changes of the NDE and also the electrooptic Kerr effect in a critical binary mixture with limited miscibility [23,37]:

(16) $$\frac{\Delta \varepsilon^{E}}{E^{2}}=\chi C{\langle \Delta M^{2}\rangle }_{V}$$

where $\Delta \varepsilon^{E}/E^{2}=\left(\varepsilon_{s}-\varepsilon_{s}\left(E\right)\right)/E^{2}$ is the NDE measure; $\varepsilon_{s}$, $\varepsilon_{s}\left(E\right)$ denote the dielectric constant and the dielectric constant under the strong electric field E; *C* is a model constant; ${\langle \Delta M^{2}\rangle }_{V}=m_{0}{\left(T-T_{C}\right)}^{2\beta }$ stands for the mean square critical contribution of the order parameter; and $\chi =\chi_{0}{\left(T-T_{C}\right)}^{\gamma }$ stands for the critical contribution of the susceptibility (compressibility) related to the order parameter.

In the supercritical region of binary mixtures with limited miscibility and one-component systems with a critical gas–liquid point, both components in Equation (13) are important. For the isotropic phase of nematogenic LC compounds, the mean-field behavior that can be associated with rod-like, uniaxial molecules is valid. In general, there are two types of pretransitional/precritical mean-field behavior [8]. Type I, often simply referred to as ‘mean-field’, is related to the space dimensionality D4 and the following values of the critical exponent, universal for any system: α=0.5 for $T=T_{C}$ and α=0 for $T=T_{C}$, β=0.5, γ=1, and ν=0.5. For the tricritical point (TCP) of type II, the dimensionality D=4 is associated with the following values of the exponents describing pretransitional/precritical point contributions to various physical properties: α=0.5 (for both $T=T_{C}$ and $T=T_{C}$), β=0.25, γ=1, and ν=0.5 [8].

For the mean-field behavior in the isotropic phase of the nematogenic LC, ${\langle \Delta M^{2}\rangle }_{V}=\Delta \varepsilon =const$, where $\Delta \varepsilon =\varepsilon_{\|}-\varepsilon_{⊥}$ is the anisotropy of the dielectric constant for the perfectly ordered sample in directions perpendicular and parallel with respect to the measured electric field. This gives rise to the relationship [23]:

(17) $$\frac{\Delta \varepsilon^{E}}{E^{2}}=\frac{\chi_{0}C^{\prime }\Delta \varepsilon }{{\left(T-T^{*}\right)}^{\gamma }}\propto \frac{1}{T-T^{*}}$$

where $T^{*}=T_{C}$ and γ=1.

Recently, pretransitional behavior has also been demonstrated in orientationally disordered crystals (ODICs) from the plastic crystal family [56]. In ODICs, translational ‘freezing’ of a crystalline network (usually cubic) is associated with the orientational freedom of permanent dipole moments tuned to the network [14,15,16,17]. The observed form of an anomaly before the transition in the liquid phase of ODICs is qualitatively different from Equation (15) but can also be described starting from the reference Equation (16), taking into account the cubic symmetry of the fluctuations before ODICs and the definition of the NDE. All this leads to the fact that the strong external electric field cannot detect changes in susceptibility associated with the uniaxial action of the strong electric field, i.e., $\chi \left(T\right)=\bar{\chi }=const$ for the ‘critical’ contribution. If one takes into account the process of the mean-field character, one obtains [56]:

(18) $${\left(\frac{\Delta \varepsilon^{E}}{E^{2}}\right)}_{crit}=\bar{\chi }C{\langle \Delta M^{2}\rangle }_{V}=c{\left(T-T^{*}\right)}^{2\beta }=c{\left(T-T^{*}\right)}^{1-\alpha }$$

Experimental evidence yielded the exponent 2β=0.5, indicating TCP-like behavior. Critical exponents are related by scaling equations so that only two exponents are independent. For the given argumentation, the following scaling relation is of importance [8]:

(19) 2−α=2β+γ

For the TCP case, the above relation has the following form [56]:

(20) 1−α=2β

Thus, taking into account the non-dipolar contribution and the non-participation in pretransitional behavior, the following relationship is obtained to describe the overall changes in the NDE in the isotropic liquid phase of ODIC-forming materials [56]:

(21) $$\left(\frac{\Delta \varepsilon^{E}}{E^{2}}\right)=\Delta \varepsilon^{*}+a\left(T-T^{*}\right)+c{\left(T-T^{*}\right)}^{1-\alpha }$$

where the exponent α=0.5.

In ODIC-forming materials, the strong electric field creates uniaxial order differences within pretransitional fluctuations and their surroundings. This leads to the differentiation between heterogeneities (fluctuations) and their surroundings, resulting in the pretransitional effects described by Equation (21). For the isotropic phase in nematogenic LC, the uniaxial, order-related distinction between fluctuations (heterogeneities) and their isotropic environment is a general system feature. To obtain changes of the type described by Equation (21), it is sufficient to determine the dielectric constant in a weak, measuring electric field, which yields the relation [56]:

(22) $$\varepsilon_{s}\left(T\right)=\varepsilon^{*}+a\left(T-T^{*}\right)+c{\left(T-T^{*}\right)}^{1-\alpha }$$

The above reasoning, which relates to distinguishing the effects of uniaxial fluctuations from the isotropic fluid environment, can also be extended to the maximum evolution of the loss curve. Remarkably, the $\varepsilon_{s}\left(T\right)\propto {\left(T-T^{*}\right)}^{1-\alpha }$ pretransitional anomaly is not present for nematogens consisting of rod-like molecules with a permanent dipole moment perpendicular to the long molecular axis. In such LC systems, the uniaxial order is preserved, but it does not lead to the cancellation of permanent dipole moments, and the difference between the dielectric constant characterizing the fluctuations and their surroundings is negligible. Nevertheless, the pretransitional effect is still detectable for the maximum primary loss curve, since the ’contrast’ between the values characterizing the fluctuations and their surroundings still exists for this order of magnitude.

The above reasoning shows the direct correlation between the pretransitional anomaly of the dielectric constant or the maximum of the primary loss curve when approaching the isotropic–nematic phase transition or related transitions in rod-like LC materials and the occurrence of local order parameter fluctuations. Parallel behavior was found when approaching the glass transition.

## 4. Conclusions

This study presents evidence of pretransitional anomalies characterized by a critical-like exponent (α=0.5) in both isotropic-to-nematic (I-N) and isotropic-to-nematic* (I-N*) phase transitions, as well as in the approach to the glass transition temperature. The similarity in the metrics for these transitions, specifically the values of $\left(\Delta T^{*}\right)$ and $\left(\Delta T_{g}^{*}\right)$, suggests these transitions have characteristics of a tricritical point. The anomalies detected are directly linked to fluctuation heterogeneities supported by the uniaxial characteristics of the rod-like liquid-crystalline (LC) systems.

Remarkably, these LC systems exhibit critical behavior for the primary relaxation time associated with the glass transition, consistent with the power exponent (ϕ≈9), aligning with the semi-heuristic dynamic glass transition model proposed by Colby and Erwin. This critical behavior is attributed to the relaxation of single molecules within a fluctuating environment before the transition. The necessity for advanced methods, such as nonlinear dielectric spectroscopy or time-resolved techniques, to directly detect the collective relaxation time $\left(\tau_{fluct}\right)$ is highlighted, given the significant challenges in studying dynamic vitrification.

This study underscores the potential of using the Ising model to represent the behavior of supercooled liquids and glassy systems, providing insights into the mechanisms controlling the glass transition through the cooperative and competitive interactions of particles. Despite the observed critical-like behavior near the glass transition sharing some notable similarities with the theoretical predictions of the Ising model and other critical phenomena theories, there are key differences that distinguish them. Both the glass transition and the Ising model exhibit features such as the divergence of correlation lengths and critical slowing down. As the system approaches the critical point—whether the glass transition temperature $T_{g}$ for glass formers or the critical temperature $T_{c}$ for the Ising model—the correlation length increases, signifying the growth of regions where molecules or spins behave cooperatively. This is accompanied by a dramatic increase in relaxation times, indicative of critical slowing down, where the system’s dynamics become markedly slower near the phase transition. However, the nature of these transitions and the underlying mechanisms differ. In the Ising model, the transition is characterized by a well-defined order parameter, typically the magnetization, which changes discontinuously at $T_{c}$ in a second-order phase transition. In contrast, the glass transition lacks a clear order parameter and is characterized by a gradual, continuous slowing down of dynamics without a sharp phase change. Additionally, the glass transition involves complex, non-equilibrium dynamics and the formation of an amorphous state, whereas the Ising model describes equilibrium critical phenomena leading to long-range order [7,8,66].

This work addresses previous difficulties in recognizing pretransitional and previtreous fluctuations due to the lack of significantly different symmetry between adjacent states around $T_{g}$. The experimental approach and choice of rod-like uniaxial molecules in this study successfully capture these heterogeneities, demonstrating long-range and tricritical pretransitional effects, a finding previously undocumented in glass transition studies.

The dynamic heterogeneities are directly responsible for the observed pretransitional anomalies near the glass transition temperature in liquid-crystalline systems. As Tg is approached, these heterogeneities manifest as critical fluctuations, causing significant anomalies in physical properties, such as the dielectric response and specific heat. The critical nature of these fluctuations, described by the critical index α = 0.5, confirms their role in the glass transition process, providing a comprehensive understanding of the mechanisms driving the transition to the glassy state. This understanding is crucial for the development and optimization of new materials with desired properties and performance near their glass transition temperatures.

## Data Availability

The raw data supporting the conclusions of this article will be made available by the authors on request.

## Abbreviations

The following abbreviations were used in this manuscript:

NDE	nonlinear dielectric effect
BDS	broadband dielectric spectroscopyu
5*CB	(S)-4-(2-methylbutyl)-4’-cyanobiphenyl
8*OCB	(S)-4-(1-methylheptyloxy)-4’-cyanobiphenyl
E7	eutectic mixture of liquid crystals
